# A novel Triclosan Methacrylate-based composite reduces the virulence of *Streptococcus mutans* biofilm

**DOI:** 10.1371/journal.pone.0195244

**Published:** 2018-04-02

**Authors:** Isaac Jordão de Souza Araújo, Andréia Bolzan de Paula, Roberta Caroline Bruschi Alonso, Jesus Roberto Taparelli, Lúcia Helena Innocentini Mei, Rafael Nóbrega Stipp, Regina Maria Puppin-Rontani

**Affiliations:** 1 Dental Materials Division, Operative Dentistry Department, Piracicaba Dental School, State University of Campinas. Piracicaba, São Paulo–Brazil; 2 Department of Dentistry, School of Dentistry, Metropolitan University of Santos (UNIMES). Santos, São Paulo–Brazil; 3 Technological Research Center, School of Dentistry, University of Mogi das Cruzes (UMC). Mogi das Cruzes, São Paulo–Brazil; 4 Department of Materials Engineering and Bioprocess, Chemical Engineering School, State University of Campinas. Campinas, São Paulo–Brazil; 5 Department of Oral Diagnosis, Piracicaba Dental School, State University of Campinas. Piracicaba, São Paulo–Brazil; 6 Department of Pediatric Dentistry, Piracicaba Dental School, State University of Campinas. Piracicaba, São Paulo–Brazil; Montana State University Bozeman, UNITED STATES

## Abstract

The use of antimicrobial monomers, linked to the polymer chain of resin composites, is an interesting approach to circumvent the effects of bacteria on the dental and material surfaces. In addition, it can likely reduce the incidence of recurrent caries lesions. The aim of this study was to evaluate the effects of a novel Triclosan Methacrylate (TM) monomer, which was developed and incorporated into an experimental resin composite, on *Streptococcus mutans* (*S*. *mutans*) biofilms, focusing on the analyses of *vicR*, *gtfD*, *gtfC*, *covR*, and *gbpB* gene expression, cell viability and biofilm characteristics. The contact time between TM-composite and *S*. *mutans* down-regulated the *gbpB* and *covR* and up-regulated the *gtfC* gene expression, reduced cell viability and significantly decreased parameters of the structure and characteristics of *S*. *mutans* biofilm virulence. The presence of Triclosan Methacrylate monomer causes harmful effects at molecular and cellular levels in *S*. *mutans*, implying a reduction in the virulence of those microorganisms.

## Introduction

Bacterial colonization, common to tooth structure, restorative materials, and prosthetic substrates [1–3], is able to modify the surfaces of these materials due to interactions with microrganisms. Metallic, ceramic, ionomeric, or resinous materials show different behaviors when submitted to bacterial aggression in vitro and in situ [4,5]. On the other hand, structure and biofilm characteristics, like glucan synthesis [6], biofilm thickness, and structure [7] could be altered by these interactions with dental materials [8,9].

Many studies have evaluated composites, because adhesive restorations are very common in clinical practice and have demonstrated some clinical longevity [10,11]. However, although resin composite restorations are extremely popular and recent advances in restorative composite technology have made the composite materials better, recurrent caries are still a reality in clinical practice and are the major cause for composite restoration replacements [12]. Recurrent caries can be due to weak bonding to dental structure, mainly on the cervical wall of class II restorations, and the interactions between the resin materials and the biofilm [13]. Consequently, the monomers used in the organic matrix of resin composites, their interactions with the biofilm formation process, and also the caries development are of interest when considering composite restorations [7,14].

Biofilm formation shows a sucrose-dependent mechanism based on glucosyltransferases (GTFs) produced by *S*. *mutans*, which combine with glucan-binding proteins (GBPs). The glucan synthesis provides for bacterial adhesion to tooth, dental materials, and other microorganisms [8]. It has been shown that by-products like Bis-HPPP, eluted from Bis-GMA, and Methacrylic Acid (MA) demonstrate inhibitory effects in the gene expression of planktonic cells [14]. In contrast, a different behavior was evidenced for gene expression analysis on biofilms when subjected to triethyleneglycol (TEG), eluted from TEGDMA, which stimulates GTFs activity and can increase the expression of some *S*. *mutans* genes [6,15]. Thus, possibly both the influence of the microorganisms on the components of the organic matrix and vice versa, may be related to the aggregation levels of the microorganisms and the elapsed time [16].

Bacterial gene expression occurs due to the importance of specific genes, mainly glucosyltransferases genes (*gtfB*, *gtfC* and *gtfD*), responsible for modulating GTF enzymes that synthesize glucans (extracellular polysaccharides)[17]. Additionally, the GBP genes, most notably *gbpB*, participate in membrane biosynthesis [18,19]. Nonetheless, the two-component transduction systems, *vicR* and *covR*, are responsible for regulating several virulence factors in *S*. *mutans* [17,20,21], including those described above.

Attempts to outline the effect of bacteria on restorative materials, particularly composites, as well as the influence of materials on biofilm characteristics, have been made, trying to reduce the virulence of *S*. *mutans* [22,23]. However, some substances added to dental materials are not used due to side effects, mainly as teeth discoloration and bacteria resistance. In addition, some products, like triclosan added as a white powder, showed a limited bioavailability when used as a polymer at 5%/wt and could not inhibit biofilm colonization over time [24]. With this perspective, the addition of new components [22,25,26] and the development of new antimicrobial monomers have been studied, when these monomers became part of the polymer chain, decreasing the degradation caused by elution of by-products and unreacted monomers [27].

Therefore, the effectiveness and safety of triclosan as an antimicrobial agent, which primarily affects the cell membrane and may even be lethal to microrganisms, depending on the concentration [28] should be considered. Accordingly, a new monomer, Triclosan Methacrylate (TM), was developed to be a part of the polymeric chain of composites. Triclosan is a trichlorinated diphenyl ether antimicrobial with one hydroxyl group. In addition, triclosan compatibilizes with a vinyl ester dimethacrylate copolymer (bis-GMA) resin and TEGDMA diluent, increasing the flexural strength. However, triclosan was previously added as a powder into the monomer, allowing its action thanks to the antimicrobial being released from the resin. Therefore, the use of a triclosan linked to a methacrylate monomer in the formulation of composites aims to reduce the effects of bacteria on material degradation, as well as reducing the potential development of secondary caries without and side effects from triclosan release [29].

The aim of this study was to evaluate the effect of a TM-containing experimental TEGDMA composite on cell viability (24h) and on the *S*. *mutans* biofilm characteristics (7 days) using quantitative measurements: average thickness, biovolume, area, and roughness surface coefficient; and qualitative measurements: cell viability (green/red) and biofilm architecture and on the expression of *vicR*, *gtfD*, *gtfC*, *covR* and *gbpB* genes (4h and 24h). The hypothesis tested was that TM-containing composite modifies the *S*. *mutans* biofilm characteristics and expression of *vicR*, *gtfD*, *gtfC*, *covR* and *gbpB* genes, decreasing the biofilm virulence. As a secondary outcome, the effect of TEGDMA-containing experimental composite was also tested concerning the expression of *vicR*, *gtfD*, *gtfC*, *covR* and *gbpB* genes on decreasing the biofilm virulence.

## Materials and methods

### Specimen preparation

The TM monomer was obtained by an esterification process of Triclosan, i.e. 2,4,4-trichloro hydroxy diphenyl ether 2 (Sigma Aldrich, St. Louis, MO, USA) with methacrylic acid in dimethylformamide (DMF) solution (Sigma Aldrich), at 30°C for 24 hours. In this study, two composites were formulated: C1 –non-antimicrobial composite; C2 –antimicrobial composite, as described in Table 1. The fillers were added incrementally and mixed homogeneously to a 50%wt loading using a high speed mixing machine (SpeedMixer^TM^ DAC 150.1 FV. Hauschild, SC, USA). When considering the filler content, the 80%wt filler was BaAlSi– 1 μm (GM27884, Schott, Landshut, Germany) and the 20%wt filler was BaAlSi– 180 nm (GM27884, Schott, Landshut, Germany). The manipulation of the experimental composites was carried out under filtered orange light.

**Table 1:** Composition of materials used in the experiments.

GROUP	MATERIAL	COMPOSITION
**CE**	CERAMIC	Leucite-reinforced glass-ceramic ingots.
**C1**	NON-ANTIMICROBIAL COMPOSITE	BISEMA^1^ 28.7%wt; TEGDMA^2^ 19.2%wt; Initiator: BAPO^3^ 2,0%wt Inhibitor: BHT^4^ 0.1%wt. Filler: Silanized Barium silicate glass 50%wt.
**C2**	ANTIMICROBIAL COMPOSITE	BISEMA^1^ 20.1%wt; TEGDMA^2^ 13.4%wt; TM 14.4%wt; Initiator: BAPO^3^ 2,0%/wt. Inhibitor: BHT^4^ 0.1%/wt. Filler: Silanized Barium silicate glass 50%/wt.

^1^Bis-phenol A ethoxylate dimethacrylate

^2^Triethylene glycol dimethacrylate

^3^Phenyl bis(2,4,6-trimethylbenzoyl) phosphine.

^4^Dibutylhydroxytoluene.

All reagents were obtained from Sigma Aldrich.

The minimum TM concentration was determined from preliminary analyzes of the antibacterial effect and the maintenance capacity of this effect, as well as based on the mechanical properties of the polymer and established at 14.4%.

Ninety-two discs (2x6mm) of C1 and C2 experimental composites were made using a silicon matrix under a laminar flow hood. The composites were inserted in the matrix and then photocured using a Bluephase photocuring unit (Ivoclar Vivadent–Schaan, Liechtenstein) at 1200mW/cm^2^ for 20 seconds.

Ceramic discs of IPS Empress Esthetic (Ivoclar Vivadent) (CE), with the same dimensions of resin discs (2x6mm), were prepared only for the gene expression analysis, as a control of the method, because ceramic materials present a bioinert behavior. After injection in an EP 600 (Ivoclar Vivadent) furnace and being removed from the coating, the discs were polished using #1200 SiC sandpaper for 30 seconds on each surface.

### Biofilm production

The discs of each material were left under UV light in a laminar flow hood for 15 minutes per surface for decontamination. Next, each disc was placed in a well of a 24-polystyrene culture plate, immersed in 1.5 ml of BHI (BD—Difco, Le Pont de Claix—FRA) with 1% sucrose inoculated with a strain of *S*. *mutans* UA159 (ATCC^®^ 700610™). Then, the discs were stored at 37°C/10% CO_2_ for 4h, 24h, and 7 days to be submitted to the analysis of gene expression (4 and 24h), cell viability (24h) and biofilm characteristics (7 days).

### Cell metabolism–XTT metabolic assay

Eight discs of C1 and C2 were used for this test. After biofilm formation on the surface of the experimental composites, the biofilm was assessed using the colorimetric method, utilizing 2,3-bis (2-methoxy-4-nitro-5-sulfo-phenyl)-2H-tetrazolium-5-Carboxanilide (XTT) to measure biofilm formation inhibition. Before the assay, the XTT solution was prepared as follows: 4 mg of XTT were dissolved in 10 ml of saline solution (37°C) supplemented with 40 μl of Coenzyme Q0. Then, after biofilm growth for 24 hours, the composite discs were washed with PBS solution to remove planktonic cells and the XTT solution was added. The discs were placed in a 24-culture well plate, which was incubated at 37°C/10% CO_2_ for 4 hours. After incubation, 200 μl of the solution was transferred to microtubes and centrifuged (13,000 rpm / 4 min) to remove residual cells and the supernatant dispensed into a 96-well plate. A microplate reader adjusted to 490 nm quantified the color alteration in the solution. This test was performed in triplicate.

### Biofilm characteristic analysis–confocal laser scanning microscopy (CLSM)

A qualitative/quantitative analysis of the biofilm characteristics was performed using CLSM. Briefly, the Live/Dead Baclight bacterial viability stain (L13152) (Molecular Probes, Eugene, OR, USA) was used. It consists of two nucleotides, SYTO 9 (stains all viable bacteria in green) and propidium iodide (stains non-viable bacteria in red). After the 7th day of biofilm growth, the non-adhering cells were removed by washing three times with saline solution. Live/Dead was mixed according to the manufacturer’s instructions and one drop of the solution was applied directly to the surface of each specimen, according to each group (n = 8). After 15 min in dark incubation, the stain surplus was removed using an absorbent paper. Non-invasive confocal images of the fully hydrated biofilms were immediately made using an inverted microscope with a CLSM unit (Leica, TCS SP5 AOBS). All images obtained were processed and analyzed by one operator, using quantitative and qualitative parameters. The quantitative analysis was made using COMSTAT software, the parameters evaluated were: biovolume (μm^3^), average thickness (μm), roughness coefficient, and surface area (μm^2^), as described by De Fúcio and cols. [30]. Descriptive parameters were used for the qualitative analysis, considering the stained areas (green and red) and dark spaces.

### Gene expression—RT-qPCR

Ninety discs of C1 (30), C2 (30) and CE (30) were made for the gene expression analysis. After biofilm growth times elapsed, the discs were removed from the culture medium and transferred to tubes with 2 ml of saline solution (0.9% NaCl). Then, the tubes were placed on vortex with 2.800rpm/10sec to detach the cells from the discs’ surface. The cells-containing solution was transferred to 2 ml micro-tubes with screw cap and O-ring (Axigen, New York—USA). The tubes were centrifuged (2 min, 4°C, ≈16000 g) for cell *pellet* precipitation and the saline solution was then discarded. The cell *pellet* was immediately stored at -80°C until RNA purification. Cell *pellet* (n = 5) was obtained from biofilms formed on four discs (4h) and two discs (24h), per group, and considered the experimental unit.

The RNA total purification was performed after breaking the frozen cells using ≈ 0.16 g of 0.1 mm diameter zirconia beads (Biospec, Bartlesville—USA), combined with 220 μl TE buffer on a Mini-bead beater apparatus (Biospec). For total RNA purification, the modified RNeasy Mini Kit protocol (50) (Qiagen, Hilden—GER) was used. The purified RNA was again frozen at -80°C for further conversion of cDNA. The RNA was then converted to cDNA using *iScript Reverse Transcriptase®* (Bio-Rad Laboratories, Hércules—USA). Reverse transcriptase reactions were prepared from a mixture containing 4 μl of *iScript®*, 60 ng of RNA from the sample, and water free DNase and RNase, and were incubated at 25°C for 5 min, 42°C for 30 min and 85°C for 5 min. In addition, to verify the absence of genomic DNA contamination of the sample, an additional reaction was prepared in the absence of *iScript®*. The converted cDNA was stored at -20°C for further analysis of gene expression.

The RT-qPCR technique was performed using a specific *primer* for the *gtfC*, *gtfD*, *vicR*, *covR* and *gbpB* genes [21]. Quantitative PCR reactions were performed on StepOne™ Real-Time PCR Systems (Applied Biosystems, UK). From each sample, 1 μl of cDNA was placed in a 48-plate well with 9 μl of a solution, containing 3.4 μl water free RNase and DNase, 0.6 μl of *primer* and 5 μl of SYBR® Green PCR Master Mix (Applied Biosystems). Standard curves (300, 30, 3, 0.3, and 0.03) were performed for each *primer* pair assay. Expression of the genes of interest was calculated and normalized by the expression of the reference 16sRNA gene.

### Statistical analysis

All data were submitted to the Kolmogorov-Smirnov test to determine the normality of the data distribution. Data from cell metabolism, surface area, biovolume, average thickness, and surface roughness coefficient, were submitted to Mann Whitney test and Wilcoxon post-test (α = 0.05). For the gene expression analysis, the data obtained from the *gtfC* gene at 24 hours were submitted to Kruskal-Wallis test with Student-Newman-Keus post-test (α = 0.05) for comparison between the groups. Data obtained from the other genes were analyzed by One-way ANOVA and Tukey test (α = 0.05) for comparison between the groups.

## Results

### Effect of TM-containing experimental TEGDMA composite resin

#### Cell metabolism—XTT

**Fig 1:**
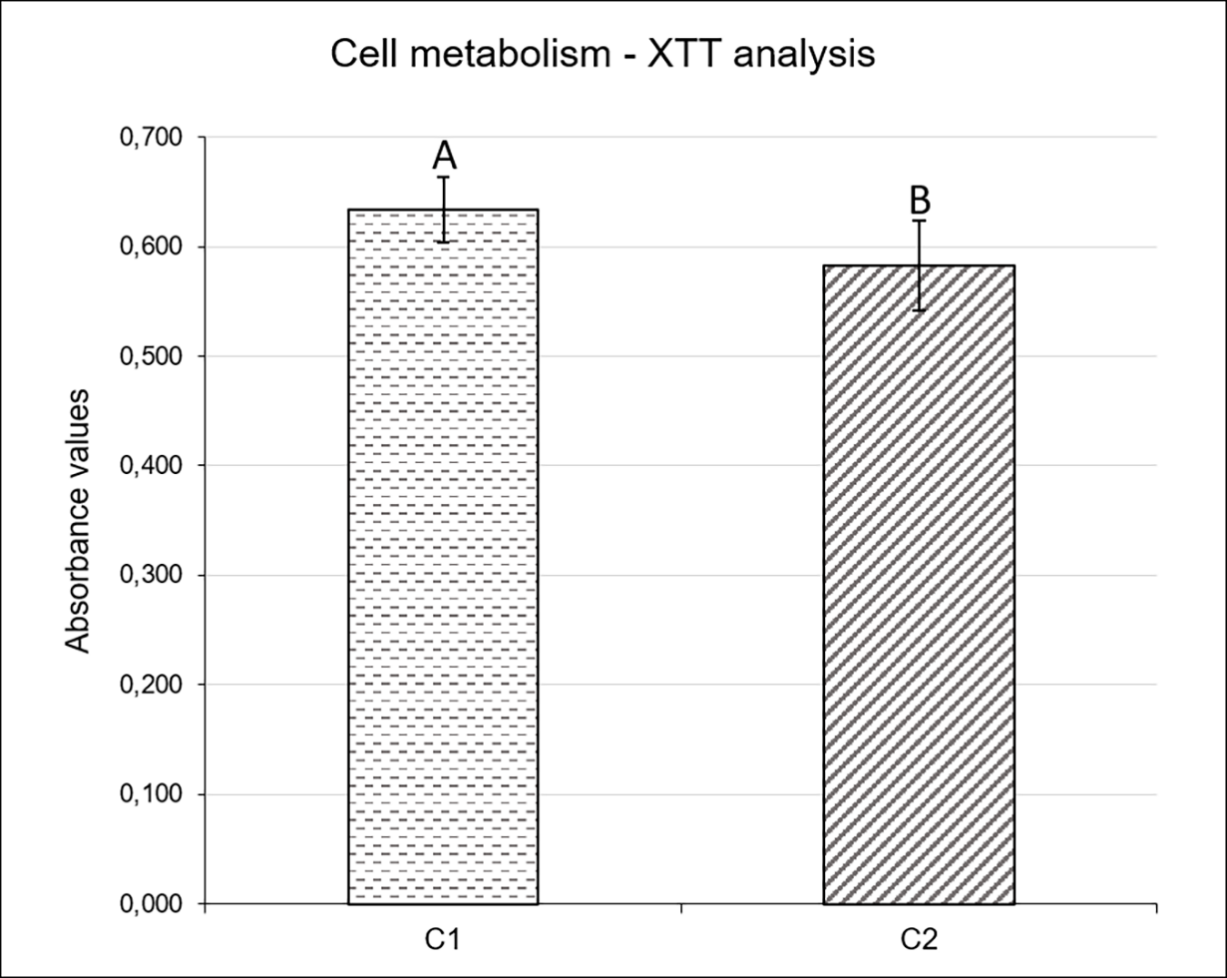
*S*. *mutans* cell metabolism *(Absorbance at 490nm)* after contact with experimental composites (C1 and C2). Different capital letters represent statistically significant differences (p< 0.05). C1- Composite resin with no antimicrobial; C2—Composite resin with antimicrobial.

Composite containing TM in its composition (C2) provided a significant reduction in cell metabolism (p = 0.0076) of *S*. *mutans* biofilms when compared to composite with no TM monomer (C1). This decrease in cell metabolism is exhibited in XTT analysis (Fig 1), whereupon C2 presented 0.582 nm (±0.041) absorbance value while C1, with no TM monomer showed 0.634 nm (±0.030).

#### Quantitative analysis of the 7th-day biofilm growth—Confocal laser scanning microscopy (CLSM)

The 7th-day biofilm growth on TM-containing composite (C2) presented significantly lower biovolume values (p = 0.028) and average thickness (p = 0.020) compared to C1, with no TM-containing composite. The roughness coefficient of the 7th-day biofilm growth on composite with no TM monomer (C1) was significantly higher than for C2 (p = 0.032). There was no significant difference on the surface area (p = 0.694) of the 7th-day biofilm growth for C1 and C2 (Table 2). Therefore, TM-containing composite provided a low virulent *S mutans* biofilm.

**Table 2:** Biovolume, average thickness, roughness and surface area values (mean and standard deviation) of the biofilm developed on restorative materials C1 and C2 for 7 days.

*Biofilm Characteristic*	*C1*		*C2*	
Biovolume (μm^3^/ μm^2^))	47.1	(27.8) A	17.6	(8.2) B
Average thickness (μm)	106.7	(52.1) A	44.1	(42.3) B
Roughness Coefficient	1.321	(0.51) A	0.799	(0.34) B
Surface area (μm^2^)	0.5343	(0.23) A	0.496	(0.14)A

Different capital letters on the same line represent statistical significant differences between C1 and C2. C1- Composite resin with no antimicrobial; C2—Composite resin with antimicrobial

#### Qualitative analysis of the 7th-day biofilm growth—Confocal laser scanning microscopy (CSLM)

**Fig 2:**
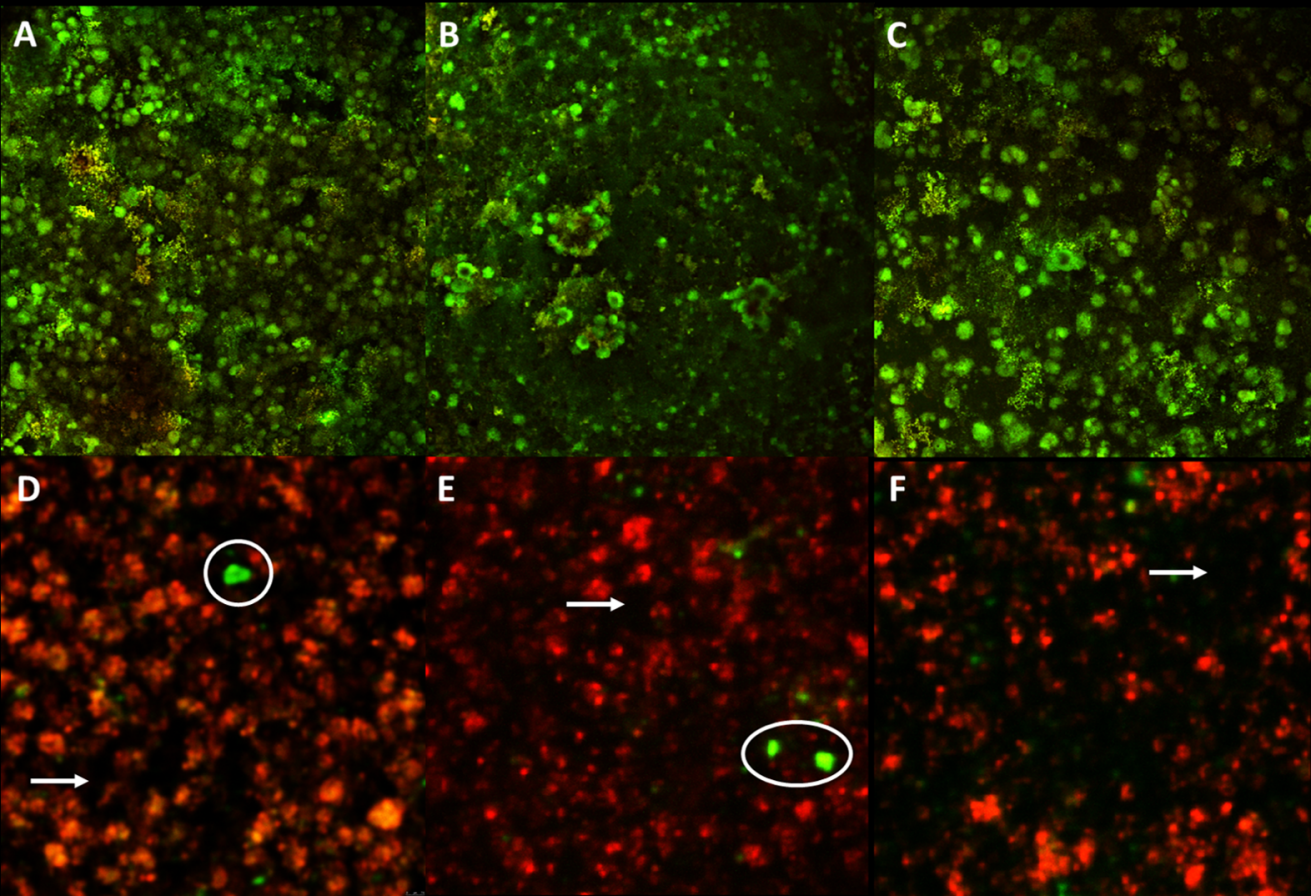
Confocal microscopy images showing the *S*. *mutans* 7-day biofilm growth on the studied materials. A, B and C: 7-day biofilm growth on C1. D, E and F: 7-day biofilm growth on C2. White arrows–voids; white circles–isle of living cells on the dead cells mass; Magnification 4x / 0.13.

CLSM analysis evidenced that TM monomer incorporated in composite (C2), provided a higher amount of unviable cells in the 7-day biofilm growth. Fig 2 shows predominant colonies of living *S*. *mutans* (green coloration) with few empty spaces (dark areas) for composite with no TM monomer (C1). In addition, 7-day biofilm growth for C2 provided the deadest cells (red staining) with scarce viable cells (circles) and evident darker areas (arrows) (Fig 2).

### Effect of TEGDMA-containing experimental composite and a TM-containing TEGDMA experimental composite

#### Gene expression—RT-qPCR

**Fig 3:**
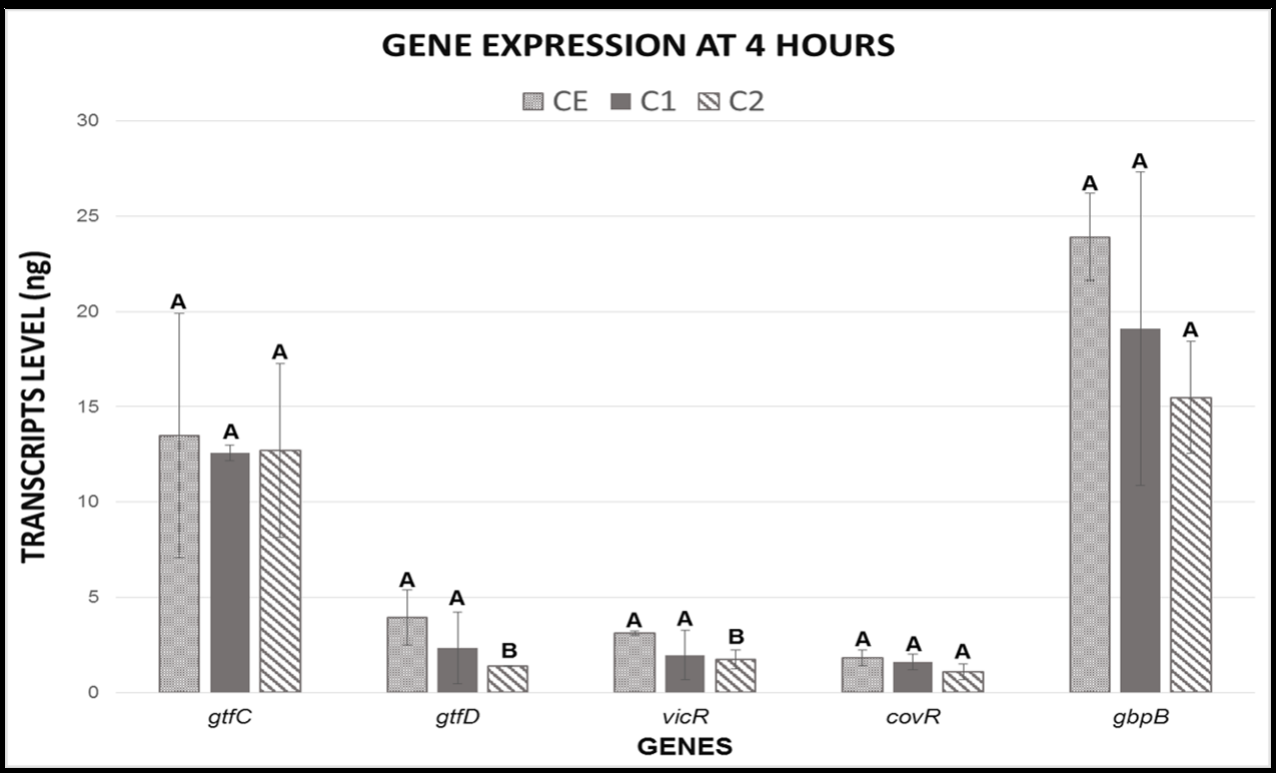
Means and standard deviations of the gene expression values in transcript levels (ng) at 4h. Different capital letters represent statistical differences between the groups for each gene analyzed individually. C1- Composite resin with no antimicrobial; C2—Composite resin with antimicrobial; CE–Ceramic.

The expressions of *gtfD* and *vicR* genes were down-regulated when the biofilm was formed in contact with TM monomer-containing composite (C2 group) at 4h, presenting a significant difference when compared to composite with no TM monomer (C1) and ceramic material (CE). This results are evidenced by the transcript level expressed in nanograms (Fig 3). On the other hand, there was no significant difference in the expression of *vicR*, *gtfD*, *gtfC*, *covR* and *gbpB* genes at 4h when compared CE and C1 (Fig 3).

**Fig 4:**
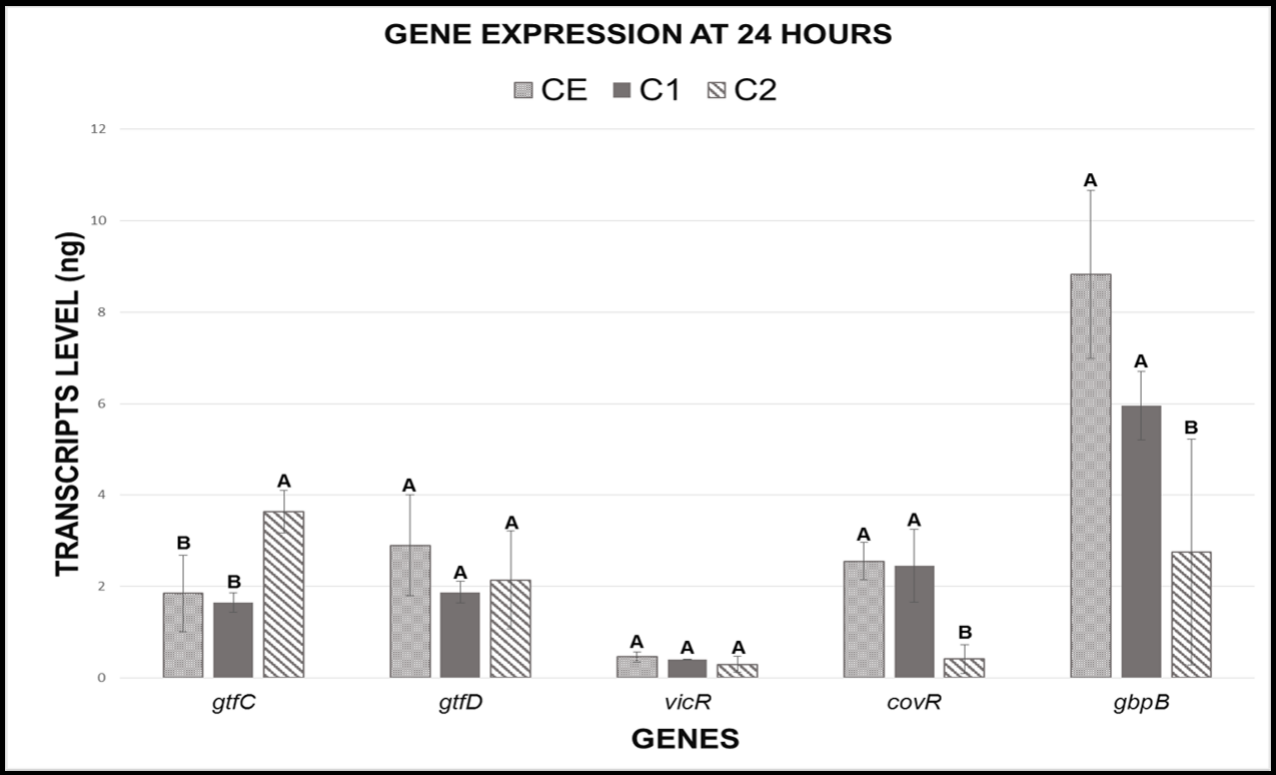
Means and standard deviations of the gene expression values in transcript levels (ng) at 24h. Different capital letters represent statistical differences between the groups for each gene analyzed individually. C1- Composite resin with no antimicrobial; C2—Composite resin with antimicrobial; CE–Ceramic.

At 24h, the composite with TM in its composition (C2 group) down-regulated the expression of *gbpB* and *covR* genes and up-regulated the expression of the *gtfC* gene when compared to composite with no TM monomer (C1) and ceramic material (CE). In contrast, as exhibited at 4h analysis, no significant differences between C1 and CE groups were evidenced for any of the genes analyzed at 24h (Fig 4).

## Discussion

Conventional resin composites present little or no antimicrobial activity and are prone to be colonized by bacteria and biofilm growth. The development of antimicrobial-containing composites has been considered and analyzed as a method for the control of secondary caries [23,31,32]. This study evaluated the effect of a TEGDMA-containing composite and an experimental antimicrobial resin composite on the development of *S*. *mutans* biofilm. The main hypothesis tested was proved that a TM-containing composite modified *S*. *mutans* biofilm characteristics and the expression of *vicR*, *gtfD*, *gtfC*, *covR* and *gbpB* genes. However, the secondary outcome shows that the TEGDMA-containing composite showed no influence on the virulence of the biofilm.

Cell metabolism data and live/dead confocal images indicate that TM can cause real interference on the *S*. *mutans* biofilm growth. A significant decrease in biovolume, average thickness, and roughness coefficient of the biofilm at the 7^th^ day of biofilm growth was demonstrated when the TM-composite (C2) was used. The changes in the biofilm structure evaluated in this study, caused by C2, reinforce the antimicrobial capacity of triclosan, even when chemically bonded to a methacrylate monomer. Therefore, the results are consistent with the idea that the use of an antimicrobial monomer as part of the polymer chain has a great perspectives for use [27].

Despite the affinity of triclosan for the wall membrane of cells [33], those biofilm structure modifications can be associated with the effect of C2 on the expression of some genes, such as v*icR*, *gtfD*, *gtfC*, *covR* and *gbpB*, since C1 showed no significant alterations on those gene expressions, similar to CE. This study showed a significant reduction on *vicR* and *gtfD* expression at 4h for C2 when compared to C1 and CE. It is likely that the expression profile of those genes was due to *gtfD* and its relationship with *vicR*. This association, between *vicR* and *gtfD*, was previously reported in UA159 strains[20], once changes in the histidine kinase sensor of *vicR* resulted in down-regulation of *gtfD* expression. The decreased expression values of these genes may indicate a reduction in *vicR*-mediated cellular functions and virulence factors, since *vicR* regulates several functions of *S*. *mutans* [19,21]. Additionally, the reduction in *gtfD*, which regulates the soluble glucan synthesis [34], may reduce the quantity of this polysaccharide in the biofilm. However, this fact was not evaluated in this study.

On the other hand, the expression of *gtfC*, *gbpB* and *covR* in *S*. *mutans* biofilms for C2 only were changed at 24h. While *covR* expression values exhibited reduction, the *gtfC* gene presented increased expression values for the TM group. This relation between *covR* activity and the expression of *gtfC* is according to the results found by Biswas and Biswas [17]. Therefore, this result, might have occurred because *covR* is an inhibitory regulator of the *S*. *mutans* virulence [17,21], which also acts on the expression of glucosyltransferases genes.

The increase on *gtfC* expression may indicate an elevation in glucan synthesis, but does not necessarily indicate an increase in virulence, since *covR* expression was decreased. The *covR* gene regulates several other virulence factors [17,35], implying alterations of the qualitative and quantitative characteristics of the biofilm, as observed in this study. Thus, a decrease in *covR* expression might have provided physiological alterations for the bacteria, which were evidenced by the reduction of viable cells (Fig 2) and the differences between C1 and C2 for biofilm roughness, thickness and biovolume (Table 2). Similar results for biofilm characteristics were found by De Fúcio and cols. [30], who showed higher roughness surface, thickness, and number of microcolonies over ceramic and ordinary composite resin discs when compared to a glass ionomer cement. In addition, our research group showed that glass ionomer cement plays some influence on the cellular activity, which was quite similar to the antimicrobial composite resin (*unpublished results*).

TM also caused a significant reduction in expression of the *gbpB* gene at 24h, which is clearly observed when C2 is compared to C1 and CE. This result may be related with the role that *gbpB* apparently plays in cell membrane biosynthesis [18,19]. Those findings reinforce the action of TM on *S*. *mutans*, since it is known that triclosan causes damaging effects on the cell membrane (Fig 2D, 2E and 2F). The *gbpB* reduction may decrease cellular co-aggregation factors, which contribute to the biofilm maturation process, since glucan-binding proteins work as an aggregation cofactor with glucosyltransferases.

The development of primary or secondary dental caries depends on a biofilm behaving as an organized microbial community [36]. Since *S*. *mutans* is one of the main agents in the carious process, the ability of the TM-composite to interfere with the biofilm by modifying the *S*. *mutans* gene expression (*vicR*, *gtfD*, *gtfC*, *covR* and *gbpB*), decreasing cell viability, and providing biofilm characteristics changes (roughness, thickness average, and biovolume decrease), can be an important tool for secondary caries control. Based on that, further *in situ* and *in vivo* studies should be conducted to verify that association.

In addition to the antibacterial activity of TM, a secondary outcome of this study was the effect of regular resin composite compounds on *S*. *mutans* biofilm. The composite without TM (C1) was unable to change the expression values of the analyzed genes or interfere with cell viability. This may indicate that by-products eluted from composites are below the minimum amount needed to cause changes to biofilm cells, which was reported by Kawai and Tsuchitani [6], and possibly closer to those reported by Polydorou and cols. [37]. The changes in bacteria are dependent of factors capable of causing changes in releasing rates of by-products, such as the presence of a filler, other matrix components and monomer ratios [38]. Although this study did not evaluate the amount of by-products eluted from the materials, the presence of BAPO as a photoinitiator may have significantly contributed to this reduction, as it allows a reduction in the amount of unconverted monomers. Therefore, BAPO would allow the formation of a more effective polymerization [39].

TM may play an important role in reducing the effects of the main agents involved in the caries processes, causing changes to bacteria at cellular and molecular levels, suggesting the real possibility of using an antimicrobial monomer in preventive and restorative dentistry.

## Conclusion

Based on the results obtained in this study, it can be concluded that:

TM-containing composite:

a decreased cell viability and changed the biofilm architecture when compared to a composite without TM;
b did not change the biofilm area, but decreased the average thickness, biovolume, and roughness surface coefficient of the biofilm at 7 days, when compared to a composite without TM;
c modified *vicR*, *gtfD* at 4-h and *gtfC*, *covR* and *gbpB* genes expression at 24h, decreasing the virulence biofilm characteristics.

Composite without TM provided no modification on *vicR*, *gtfD*, *gtfC*, *covR* and *gbpB* at expression at 4h and 24h.
